# CHANGES IN LIFE-SPACE WITH LOSS OF RELATIVES AND FRIENDS AMONG OLDER ADULTS: RESULTS FROM THE UAB STUDY OF AGING

**DOI:** 10.1093/geroni/igac059.2137

**Published:** 2022-12-20

**Authors:** Amelia Driggers, Richard Kennedy, David Buys

**Affiliations:** Mississippi State University, Mississippi State, Mississippi, United States; University of Alabama at Birmingham, Birmingham, Alabama, United States; Mississippi State University, Mississippi State, Mississippi, United States

## Abstract

Increasing age brings greater risk of the death of friends and family (hereafter referred to as loss). Loss may impact individuals’ life-space mobility (LSM); however, no research has explored relationships between loss and LSM trajectories. Using the UAB Study of Aging, we examined differences in LSM trajectories of 1000 community-dwelling older Alabamians (65+years) with and without loss during the study period. We assessed LSM using the UAB Life-Space Assessment (LSA), a validated instrument measuring individuals’ ability to move through zones ranging from their bedroom to out of town. We assessed loss every 6 months using a standard bereavement questionnaire capturing spousal, other relative, or friend loss. We used piecewise linear mixed effects models to compare LSA trajectories. Those who do not experience loss had a baseline mean LSA score of 49.5 and show a decline of 0.08 points per year (p < 0.001). Those that do experience loss had a baseline LSA score of 60 and decline by 1.0 point per year before the loss (p < 0.001), accelerating to 1.8 points per year after the loss (p < 0.001). Those with loss do not experience acute decline post loss but do have an acceleration of the pre-existing decline. More research is needed on this topic to better understand the impact of loss on LSM trajectories; but this finding suggests that more public health and clinical interventions may be needed for those who experience loss. Specifically, bereaved individuals may benefit from social, mental, or healthcare services for loss-related challenges.

